# Prolific

**Published:** 1858-01

**Authors:** A. Garwood

**Affiliations:** Cassapolis, Mich.


### PROLIFIC.

CASSAPOLIS, MICH., Jan. 12th, 1858.

PROF. N. S. DAVIS, CHICAGO:

DEAR SIR,—A colored lady of this county gave birth on the 4th inst. to four children—three boys and one girl—weighing four and a quarter pounds each; mother and children doing well. These make seven children to which she has given birth in less than three years. At her first confinement she had one, at the second two, and the last four. They are very poor, and there has been a subscription circulated to buy them a cow, but if they continue to increase in geometrical progression, they will soon need quite a dairy.

A. GARWOOD, M. D.

